# Comprehensive Analysis of Immune-Related Metabolic Genes in Lung Adenocarcinoma

**DOI:** 10.3389/fendo.2022.894754

**Published:** 2022-07-08

**Authors:** Fangfang Li, Chun Huang, Lingxiao Qiu, Ping Li, Jiang Shi, Guojun Zhang

**Affiliations:** Department of Respiratory Medicine, The First Affiliated Hospital of Zhengzhou University, Zhengzhou, China

**Keywords:** lung cancer, immune, metabolic, TCGA, immunotherapy

## Abstract

**Purpose:**

The immunotherapy of lung adenocarcinoma (LUAD) has received much attention in recent years and metabolic reprogramming is linked to immune infiltration in the tumor microenvironment. Therefore, it is indispensable to dissect the role of immune-related metabolic genes in lung adenocarcinoma.

**Methods:**

In this study, we screened immune-related genes by Pearson correlation. The function of these genes was explored by gene ontology (GO) and KEGG (Kyoto Encyclopedia of Genes and Genomes) enrichment analysis. The differently expressed immune-related genes were analyzed by Limma. Furthermore, the LUAD patients were clustered based on immune-related genes through consensus clustering. The Unicox was used to identify survival-immune-related metabolic genes. The Least Absolute Shrinkage and Selection Operator (LASSO) regression analysis was used to optimize the gene sets. A prediction model was constructed and tested. The potential therapeutic target was selected based on two criteria, these immune-related metabolic genes that were highly expressed in tumor tissues and negatively correlated with the survival of patients in LUAD. Quantitative real‐time PCR (qRT‐PCR) was used for *in vitro* experimental validations.

**Results:**

We identified 346 immune-related genes, mainly involved in arachidonic acid metabolism and peroxisome proliferator-activated receptor (PPAR) signaling. Moreover, a total of 141 immune-related genes were dysregulated between tumor and normal tissues. We clustered three subtypes of LUAD based on immune-related metabolic genes and these subtypes exhibited different survival and immune status. We found Ribonucleotide Reductase Regulatory Subunit M2 (*RRM2*) as a potential therapeutic target, which is positively correlated with the cyclin-dependent kinase family of genes.

**Conclusion:**

We comprehensively analyzed the immune-related metabolic genes in LUAD. *RRM2* was determined as a promising metabolic checkpoint for lung adenocarcinoma.

## Introduction

Lung cancer is one of the most common causes of cancer-related mortality. Adenocarcinoma is the most common histological type of lung cancer (1, 2). Lung adenocarcinoma (LUAD) has an unfavorable 5-year survival rate which makes only 15% (3–5). In the past few decades, surgical resection, chemotherapy, radiotherapy, and targeted molecular therapies have been carried out in clinical practices to treat LUAD. However, most LUAD patients are usually diagnosed at advanced and late stages, thus having poor prognosis. In recent years the relationship among cancer immunotherapy, tumor microenvironment, and metabolism has gotten much of attention. Hence, comprehensively understanding the role of immune-related metabolic genes involved in the occurrence and development of LUAD is crucial for the diagnositc and prognositic prospetcs.

The tumor microenvironment (TME) is the cellular environment in which the tumor develops. TME is closely related to the occurrence and development of tumors (6, 7). It included inflammatory and stromal cells that infiltrate the tumors. Lymphocytes infiltrating tumor tissues have been discovered for more than hundred years. After 1960, people began to consider the relationship between immunity and prognosis (8). It has been found that the infiltration of T cells (80%) in the majority of tumors is positively correlated with the tumor metastasis (9). Aberrant cellular metabolism is emerging as a novel therapeutic target, and the interplay between metabolic remodeling and immune regulation in cancer represents a potential area of investigations (10, 11).

Abnormal activation of oncogenic genes, such as Myc and Ras can directly regulate intracellular metabolic pathways (12). Moreover, immune cells can also change metabolic pathways and further affect cellular functions (13). The abnormal metabolism of tumors not only enables tumors to survive in an environment of hypoxia and nutrient deficiency, but the products of metabolism can inhibit immune response, promote the formation of immunosuppressive cells, and help tumors evade host immune killing (14). It has been found that in acute lymphoblastic leukemia, proliferating T and B cells exhibit abnormal metabolic stress (15, 16). Similarly, mounting evidence has confirmed that reprogramming the tumor immune microenvironment is a necessary process that drives LUAD metastasis (17). This suggests that the metabolic disorder of cancer cells may be treated by targeting some genes (18).

In this study, we identified 346 immune-related genes. Among these, 141 genes were found to be dysregulated between normal and tumor tissues. Three clusters of LUAD samples were based on immune-related metabolic genes and different clusters exhibited distinct survival and immune status. Moreover, we constructed and validated a prediction model and identified *RRM2* as a potential metabolic target which was positively correlated with the cyclin-dependent kinase (CDK) family of genes.

## Materials and Methods

### Data Preprocessing

The mRNA sequencing and clinical data of 535 LUAD samples and 59 normal samples were downloaded from the TCGA data portal. The metabolism-related genes were downloaded from published work (19). The immune-related genes were downloaded from an online website (https://www.immport.org/). Low expressed genes were excluded from the study and the data was normalized to log2 (tpm+1) (average expression after normalization <0.5). Finally, 346 immune-related metabolic genes were selected by cor test using the Pearson correlation method (P<0.05, |R|>0.2).

### GO and KEGG Enrichment Analysis and PPI Network Construction of Immune-Related Metabolic Genes

We divided 346 differentially expressed genes (DEGs) into up-regulated and down-regulated genes. R was used to perform GO and KEGG enrichment analysis. The “clusterProfiler”, “richplot”, and “ggplot2” packages were used for analysis (20, 21). The GO analysis was performed to annotate genes and classify up-regulated and down-regulated DEGs. The GO terms consisted of 3 parts: Biological Process (BP), Cellular Component (CC), and Molecular Function (MF). The KEGG database included the systematic analysis, annotation, and visualization of gene functions (22). STRING online website was used to construct a protein-protein interaction (PPI) network for the selected DEGs (23). For PPI analysis, the confidence score was set to > 0.9, and only terms with both p- and q-value of <0.05 were considered significantly enriched. Cytoscape software further analyzed the most closely connected modules and identified the top 10 central genes (24).

### Identification of Dysregulated Genes Between Tumor and Normal Tissues

The “limma” package (25) in R was used to identify DEGs between Cancer and adjacent tissue samples. Merely genes with | log2fold change | > 1 and P < 0.05 were considered as DEGs. The “pheatmap” package was used to draw heat maps, and “ggplot2” was used to draw volcano maps.

### Consistent Clustering of Immune-Related Metabolic Genes

The immune-related metabolic genes were divided into different clusters by the cell consistency clustering method. We used the “ConsensusClusterPlus” package (100 iterations and 80% resampling rate, http://www.bioconductor.org/) to classify patients with LUAD into different subtypes. The heat map and dela diagram established the optimal number of clusters. The cumulative distribution function (CDF) was plotted to identify the number of best clusters. The Progress Free Survival (PFS) between various clusters was compared. The survival analysis was analyzed by the R package “survival”, and the “ggplot2” package was used for plotting.

### Immune Characteristics Between Clusters-Expression of Immune-Related Molecules

The expression of immune-related molecules among these clusters with the ESTIMATE algorithm was analyzed by the R “ESTIMATE” package. These immune-related genes regulate four immune functions these included, antigen presentation, chemokine-related genes, cytokines, and immune checkpoints. “ggplot2” package was used to draw box plots.

### Immune Characteristics Between Clusters-Expression of Infiltrating Immune Cells and Clinicopathological Characteristics

Four methods were used to assess the infiltration of immune cells in three clusters. These methods were single-sample gene set enrichment analysis (ssGSEA), Microenvironment Cell Populations (MCP)-counter, CIBERSORT, and Xcell (26–29). The three different immune cell infiltrating clusters were also compared and found their immune scores. We compared the pathological classification proportions between different clusters to further distinguish the differences between different clusters, including T, N, M, clinical drug treatment response, and the pathological stage.

### Validation of Prognostic Prediction Based on Immune-Related Metabolic Genes Models

The expression matrix of LUAD was randomly divided into training and test sets. The training set was 70% and the test set was 30%. We used single-factor analysis on the two groups of genes. The genes with p<0.05 were selected. The Least Absolute Shrinkage and Selection Operator (LASSO) cox regression method was further optimized through multi-factor COX regression analysis to help us to determine the best number of genes to build a model (30, 31). Moreover, we collected 80 lung cancer samples along with complete survival information and constructed a prognostic model as a control. Finally, the gene’s risk score was screened to get a good predictive ability on the patient’s survival. The area under the ROC curve (AUC) was used to judge the prognostic model’s predictive power. The ten-fold cross-validation based on the “glmnet” package in R was used for lasso penalty Cox regression analysis. The survival analysis was analyzed by R package “survival”, while AUC was analyzed by R package “survivalROC”.

### Identification of Potential Metabolic Checkpoints

First we selected the immune metabolism genes that were highly expressed in the tumor site (logFC>1.5, FDR<0.05), and the immune metabolism genes in the training set that were negatively related to survival (p ≤ 0.01). Through pan-cancer analysis (https://cistrome.shinyapps.io/timer/) and the expression level analysis of collected clinical samples, we screened out genes with higher expression abundance in tumors. The signaling pathway was determined with criterion (spearman r>2 or<-2, q value< 0.05) using online website cBioPortal (https://www.cbioportal.org/). The selected genes were used to perform GO and KEGG enrichment analyses.

### Cell Culture

The different cell lines used in this study were, Human normal lung epithelial cell line (BEAS-2B) A549, NCI-H292, and Calu-3. BEAS-2B cells were purchased from Procell Life Science and Technology Co., Ltd. (Wuhan, China). A549, NCI-H292, and Calu-3 were purchased from the cell bank of the Chinese Academy of Sciences (Shanghai, China). The BEAS-2B cell line was cultured in Dulbecco’s Modified Eagle’s medium (DMEM; Gibco, Grand Island, NY, USA). The A549 cell line was cultured in Ham’s F-12K medium (Gibco) The NCI-H292 cell line was cultured in Roswell Park Memorial Institute-1640 medium (RPMI-1640; Gibco), and the Calu-3 cell line was maintained in modified eagle medium (MEM; Gibco). All media were supplemented with 10% fetal bovine serum (FBS; Gibco) and antibiotics (100 units/ml penicillin and 100 ug/ml streptomycin; Gibco). All cells were incubated in a humidified atmosphere of 5% CO2 at 37°C.

### Quantitative Real‐Time PCR

Total RNA was isolated from tissues and cells using TrIzol reagent (Gibco) according to the manufacturer’s instructions. The extracted RNA was reverse transcribed into complementary DNA using a reverse transcription kit (Takara, Dalian, China). Quantitative real-time PCR (qRT-PCR) was performed using the SYBR-Green PCR kit (Roche Diagnostics, Indianapolis, IN) on a Step One Plus Real-Time PCR system (Applied Biosystems, Foster City, CA). Glyceraldehyde 3-phosphate dehydrogenase (*GAPDH*) was used as an internal control. The results were analyzed using the 2^-ΔΔCt^ method. Primers were synthesized by Sangon Biotech (Shanghai, China). All the primer sequences were listed (**Supplementary Table 1**).

## Results

### Identification and Function Enrichment Analysis of Immune-Related Metabolic Genes

**Figure 1:**
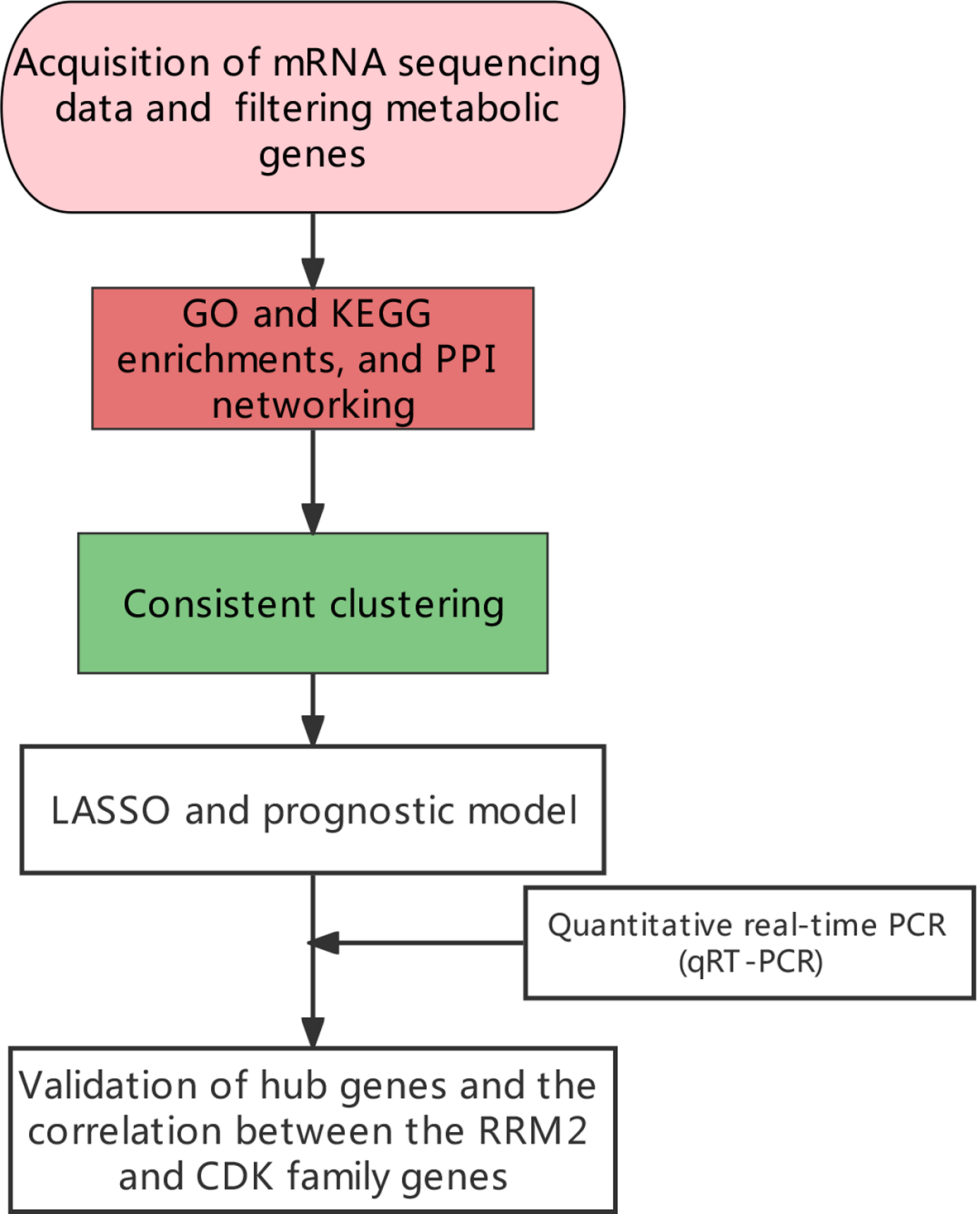
The general research design and flow of the study.

**Figure 2:**
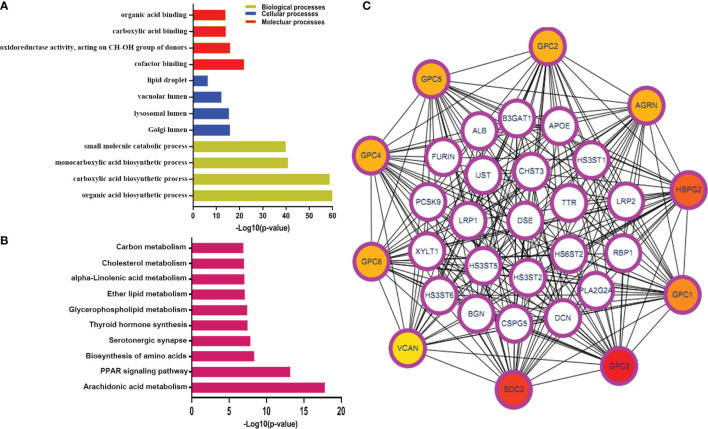
The GO and KEGG pathway analysis for immune-related metabolic genes. **(A, B)** The GO enrichment and KEGG pathway analyses of immune-related metabolic genes; **(C)** The top 10 genes were ordered by the number of nodes. BP, biological process; CC, cellular component; MF, molecular function; KEGG, Kyoto Encyclopedia of Genes and Genomes.

To identify the immune-related metabolic genes, we obtained 1041 immune genes and 1613 metabolic genes. The general research design and flow of the study was shown (**Figure 1**). The correlation analysis identified 346 immune-related metabolic genes (**Figure S1**). The GO analysis consisted of three parts: BP, CC, and MF. Our results indicated that the immune-related metabolic genes were significantly enriched in the BP-associated organic acid biosynthetic process, carboxylic acid biosynthetic process, and monocarboxylic acid biosynthetic process. For the CC, the immune-related metabolic genes were mainly enriched in the Golgi, lysosomal, and vacuolar lumens. Furthermore, the MF analysis showed that the immune-related metabolic genes were significantly enriched in cofactor binding, oxidoreductase activity, acting on the CH−OH group of donors, and carboxylic acid-binding. The immune-related metabolic genes were found to be involved in Arachidonic acid metabolism, PPAR signaling pathway, and Biosynthesis of amino acids (**Figures 2A, B**). PPI network was established to further dissect the potential mechanism of these genes (**Figure 2C**). Top10 core genes were identified by Cytoscape plug-in cytoHubba: these genes included, *SDC2*, *GPC3*, *GPC1*, *HSPG2*, *AGRN*, *GPC2*, *GPC5*, *GPC4*, *GPC6*, and *VCAN* (**Figure 2C**).

The heat map clearly distinguished the immune related metabolic genes in tumor and normal tissues (**Figure S2A**). A total of 141 DEGs were identified (|log2fold change |> 1, P < 0.05). Among these, 72 genes were up-regulated and 69 genes were down-regulated (**Figure S2B**). Then, we performed GO and KEGG enrichment analysis on 141 differential genes (**Figures S2C–F**). It was observed that DEGs were mainly involved in the fatty acid metabolic process; organic hydroxy compound metabolic process and small molecular metabolic process.

### Consistent Clustering of Immune-Related Metabolic Genes

**Figure 3:**
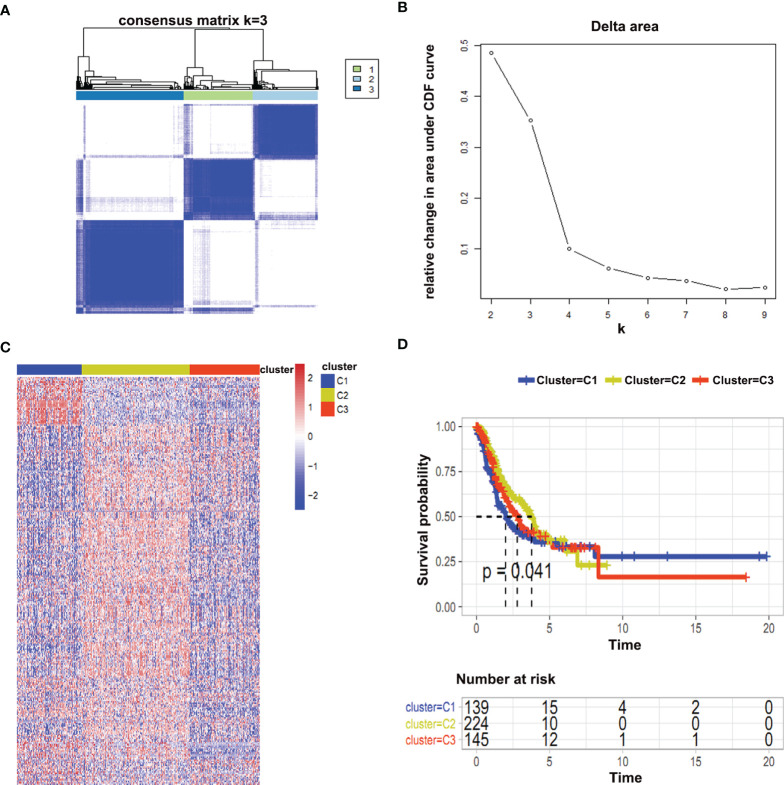
Consistent clustering of immune-related metabolic genes. **(A, B)** Cumulative distribution function (CDF) represented an optimal number of clusters (k is 3); **(C)** Heat map represented immune-related metabolic genes in three clusters; **(D)** Survival analysis between different clusters was shown.

Consistent clustering of immune-related genes was performed to unwind metabolic patterns of tumor cells. Tumor samples were divided into different clusters according to the expression patterns of immune-related gens. To determine the optimal cluster number, the cumulative distribution function (CDF) was plotted and three different clusters were identified. Moreover, heat maps were drawn to compare the expression of immune-related metabolic genes among the various clusters (**Figures 3A–C**). Furthermore, the survival status of the three clusters was evaluated by comparing progression-free survival (PFS) and clinicopathological parameters. Our results showed that cluster 2 have prolonged survival in early times (**Figure 3D**). Consistent with these findings, patients in the cluster 2 have lower T, N, M, and stage as well as the status of lymph node metastasis (**Figures S3A–D**). Similarly, a comparatively more proportion of patients had a complete response to treatments in this cluster (**Figure S3E**).

### Immune Characteristics of the Three Different Clusters

**Figure 4:**
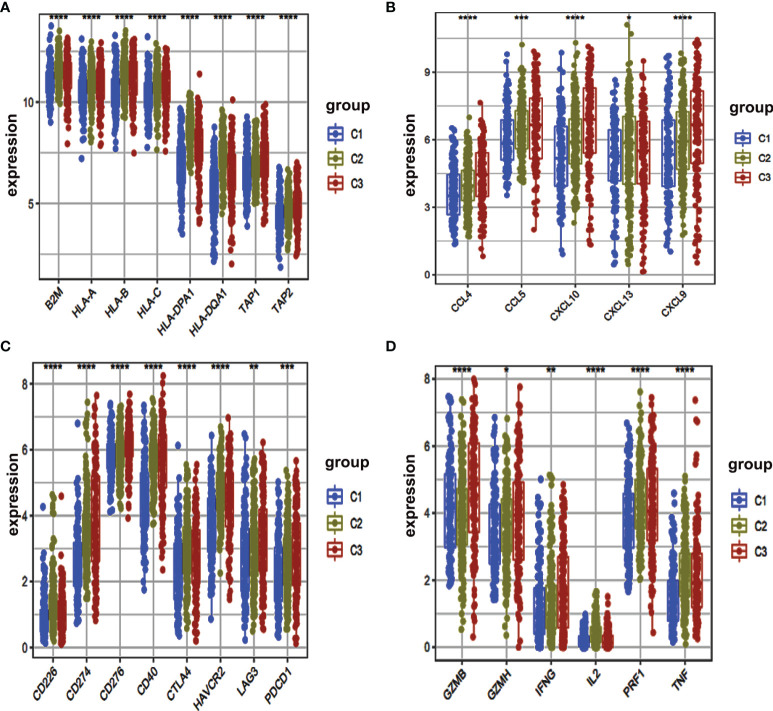
Box plot of immune-related gene expressed among different clusters. **(A–D)** The expression levels of multiple immune genes were compared in three clusters. *, P < 0.05. **, P < 0.01. ***, P < 0.001. ****, P < 0.0001.

Furthermore, we were interested to determine immune-related genes in these clusters. We determined those immune-related genes that were involved in antigen‐presentation (*B2M, HLA-A, HLA-B, HLA-C, HLA-DPA1, HLA-DQA1, TAP1, TAP2*), chemokine-related genes (*CCL4, CCL5, CXCL10, CXCL13, CXCL9*), immune checkpoint genes (*CD226, CD274, CD276, CD40, CTLA4, HAVCR2, LAG3, PDCD1*) and genes responsible for the production of cytokines (*GZMB, GZMH, IFNG, IL2, PRF1, TNF*) expressions. We used a box plot for comparison and found that *HLA-DPA1, HLA-DQA1, HLA-B, CXCL13 and CD226* had a high expression within C2 cluster (**Figures 4A–D**).

**Figure 5:**
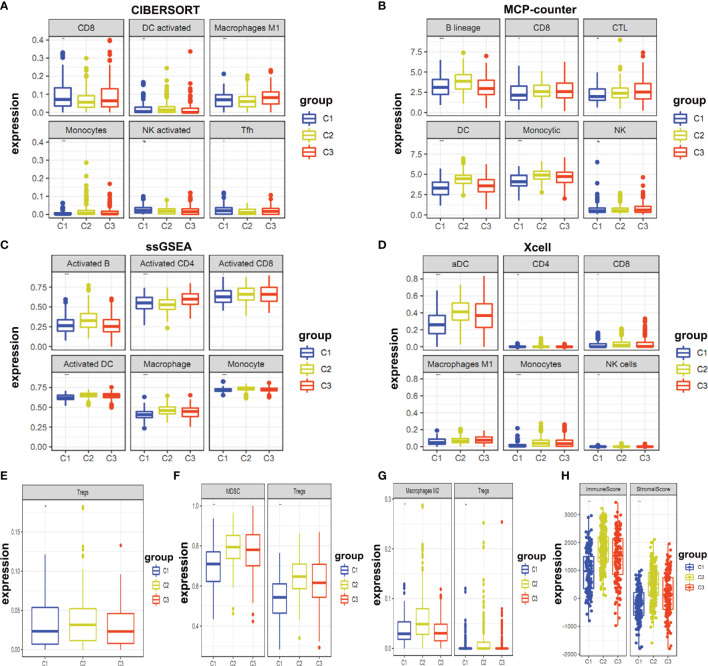
Expression levels of immune infiltration cells. **(A–D)** Expression levels of immune effector cells between different clusters were shown; **(E–G)** Immunosuppressive cells in different clusters were shown; **(H)** Immune and stromal scores in different clusters were shown.

Moreover, we determined infiltration levels of immune cells in these clusters. We used four reported methods (CIBERSORT, MCP-counter, ssGSEA, and Xcell) for this purpose. Two aspects were explored for these clusters i.e. immune effector cells (**Figures 5A–D**) and immunosuppressive cells (**Figures 5E–G**). Our analysis delineated that Cluster 1 had the least infiltration of immune effector cells and immunosuppressive cells (**Figures 5A–G**). This suggests that cluster1 might be the immunologically-cold tumors. Cluster 2 and cluster 3 were found to be enriched in the immunologically-hot tumor immune microenvironment. Both of these clusters were enriched in both immune effector and immunosuppressive cells. Activated B cells, dendritic cells (DC), and monocytes were significantly enriched in cluster 2 (**Figures 5A–G**). We compared the three different clusters and reached their immune score. The results showed that cluster 2 had higher immune and stromal scores (**Figure 5H**).

### Construction of Prognostic Prediction Models of Immune-Related Metabolic Genes

**Figure 6:**
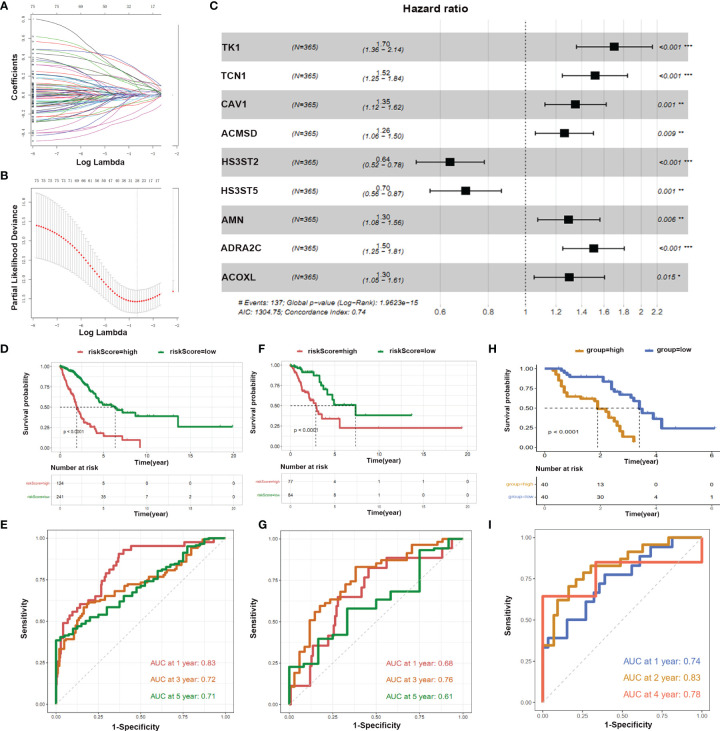
LASSO and hub genes of prognostic model. **(A, B)** Optimal values of the penalty parameter λ; **(C)** Multivariate regression analysis of nine genes we shown; **(D, F, H)** OS in the low score group was significantly higher than in the high score group; **(E, G, I)** Time-dependent ROC curves analysis of the prediction model.

Next, we were interested in whether these immune-related metabolic genes could be used to predict survival. The LUAD matrix was divided into training (70%) and test (30%) sets. We selected 80 genes having p<0.05 and performed Unicox analysis to compute the regression coefficient (**Figures 6A, B**). Moreover, multivariate regression was performed to calculate the formula. We identified nine genes that were used to construct a prediction model. These nine genes were, *TK1, TCN1, CAV1, ACMSD, HS3ST2, HS3ST5, AMN, ADRA2C, and ACOXL* (**Figure 6C**). Patients were categorized into high and low-risk groups in training and test sets. A survival curve was plotted according to the clinical information of two groups of patients (**Figures 6D–F**). The results showed that training and test sets with high-risk score patients had a worse Overall Survival (OS) rate than those of low score patients (p <0.0001) (**Figures 6D–F**). The area under the ROC curves of the predictive model for LUAD has the same performance in the first year, third year, and fifth-year (Training set: AUC at one year: 0.83, AUC at three years: 0.72, AUC at five years: 0.71; Test set: AUC at one year: 0.68, AUC at three years: 0.76, AUC at five years: 0.61) (**Figures 6E–G**). Moreover, the prediction model was validated using our clinical specimens. The validation results confirmed that the high-risk score group had a worse survival (p <0.0001) (**Figures 6H, I**). Meanwhile, our prediction model had high accuracy (AUC at one year: 0.74, AUC at three years: 0.83, AUC at four years: 0.78).

### Identification of Potential Metabolic Targets

**Figure 7:**
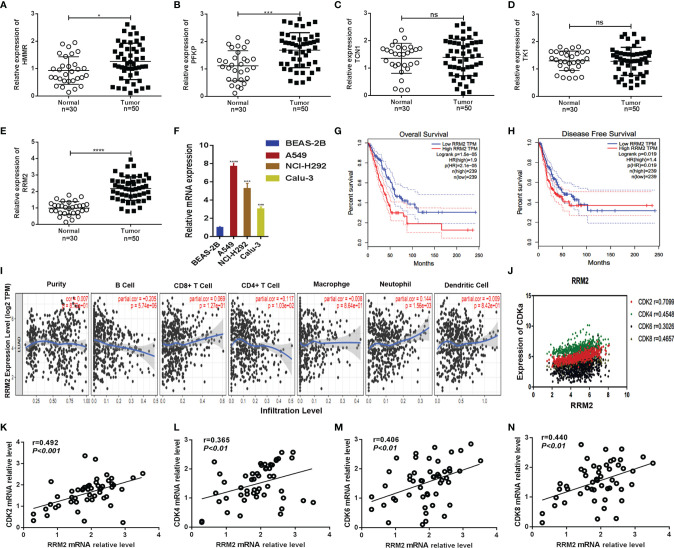
*RRM2* was related to survival time and CDK family. **(A–E)** Expression levels of *HMMR, PFKP, TCN1, TK1*, and *RRM2* in non-cancerous tissues (n = 30)and LUAD tissues (n=50) were detected by qRT-PCR. *GAPDH* was used as an internal control; **(F)** *RRM2* expression levels in different cell lines were determined by qRT-PCR. *GAPDH* was used as an internal control; **(G, H)** Kaplan–Meier analysis was performed to assess the association of *RRM2* expression with overall survival and disease-free survival in LUAD patients using the TCGA databases; **(I)** Correlation of *RRM2* with immune cell infiltration in lung cancer; **(J–N)** Association of *RRM2* with the CDK2 family of proteins. *, P < 0.05. **, P < 0.01. ***, P < 0.001. ****, P < 0.0001. ns : Not Statistically Significant.

As the highly expressed genes in the tumor could be a potential factor to promote tumor growth, therefore, we selected those immune-related metabolic genes whose expression was high in the tumor site (logFC > 1.5, FDR < 0.05). Five potential targets i.e., *HMMR, PFKP, RRM2, TCN1*, and TK1 were obtained that were negatively correlated with survival rate (**Figure S4**). The expression of five hub genes in pan-carcinoma was shown (**Figure S5**). The correlation of the four genes *HMMR, PFKP, TCN1*, and *TK1* with tumor-infiltrating immune cells and the survival curve in lung cancer was shown (**Figure S6**). Furthermore, we determined the expression levels of five hub genes in our clinical specimens. We found that the expression of *RRM2* was higher in tumor tissues (**Figures 7A–E**). Similarly, the expression level of *RRM2* was significantly higher in lung cancer cell lines (A549, NCL-H292, and Calu-3) compared to normal lung epithelial cell line (BEAS-2B) (**Figure 7F**). The survival curves of *RRM2* and immune cell infiltration in lung cancer patients were determined. The overall survival of lung cancer patients showed that low *RRM2* expression had a better prognosis (p=0.000015) (**Figure 7G**), and disease-free survival also suggested that patients with low *RRM2* expression had a better prognosis (p=0.019) (**Figure 7H**). The relationship with immune cells showed that *RRM2* was associated with tumor infiltration by B cells and Neutrophils (**Figure 7I**).

To determine the function of *RRM2*, GO and KEGG analyses were performed to find correlated genes with *RRM2* (**Figure S7**). The *RRM2*-related genes were mainly enriched in catalytic activities, acting on DNA as determined by GO analysis. While KEGG pathway enrichment analysis showed that *RRM2*-related genes were mainly enriched in cell cycle regulation. Moreover, the correlation between the *RRM2* gene and the CDK family of genes was analyzed. This result showed that the *RRM2* gene was highly related to the CDK family of proteins (**Figure 7J**). We also found the same results in tumor samples analyzed by qPCR, showing that this gene was associated with CDK family of proteins. Our results delineated that the expression levels of *RRM2*, *CDK2* (r=0.492, p<0.001), *CDK4* (r=0.365, p<0.01), *CDK6* (r=0.406, p<0.01) and *CDK8* (r=0.440, p<0.01) were positively correlated, which means that *RRM2* was significantly correlated with cell cycle signaling (**Figures 7K–N**).

## Discussion

Our study identified five potential metabolic checkpoints of LUAD and *RRM2* was chosen for further analyses. The expression of RRM2 was significantly higher in both lung cancer tissues and cell lines. In the current study, we disseminated the possible pathways regulated by *RRM2* in lung cancer. We further showed that the cell cycle could be regulated by *RRM2*.

Tumor microenvironment infiltration is closely related to immunotherapy effectiveness. The critical role of immune-related metabolic genes and immune cells in cancer is gradually being unveiled. Therefore, we were interested to find immune-related genes that play a role in immune infiltration and could produce a better immunotherapy effect. In our study, the immune-related genes were obtained from the website, https://www.immport.org/. The metabolism-related genes were downloaded from work published by Peng, X. We performed different analyses and identified ten immune-related metabolic genes. These genes were, *SDC2, GPC3, GPC1, HSPG2, AGRN, GPC2, GPC5, GPC4, GPC6, and VCAN*. These genes play important roles in the immune-related mechanisms of several cancers (colorectal (32), cervical (33), liver (34), pancreatic cancer (35), etc). Immune infiltration of tumor microenvironment in glioblastoma multiforme, breast cancer, and lung cancer play a vital role in immunotherapy and the increase in the degree of immune infiltration is related to better immunotherapy effect (36–39).

To explore the specific mechanisms of these immune-related metabolic genes, the samples were divided into three clusters. The levels of immune cell infiltration, immune scores, and clinicopathological information were compared. We found that among all clusters, cluster2 had prolonged survival at the early stages of the disease. *HLA-DPA1, CXCL13*, activated B cells, DC, and monocytes infiltration were highly expressed in cluster 2. *HLA-DPA1* is involved in immune responses and antigenic peptides presentation (40). Previous studies demonstrated that down-regulation of *HLA-DPA1* expression is related to the poor prognosis of tumors and may be a potential prognostic biomarker for ESCC (41–43). Therefore, higher expression of *HLA-DPA1* in cluster 2 could well represent the prolonged survival of LUAD patients. The *CXCL13*/*CXCR5* signal axis plays a vital role in the occurrence and development of several human cancers (44). The prognosis was found better in cluster 2 compared to cluster1 and cluster3. The pathways related to B cells play important role in tumor immunotherapy (45, 46). Similarly, monocytes also play an important role in antigen presentation in the microenvironment of tumor immune infiltration (47, 48).

Furthermore, nine genes *TK1, TCN1, CAV1, ACMSD, HS3ST2, HS3ST5, AMN, ADRA2C*, and *ACOXL* were identified for the construction of prediction model. Our findings are parallel with previous findings. *TK1, TCN1, CAV1, and HS3ST2* play indispensable roles in survival predictions and pathogenesis of various cancers (49–55).

Finally, we obtained five potential metabolic checkpoints of LUAD. These were, *HMMR, PFKP, RRM2, TCN1* and *TK1*. By comparing their expression levels and their association with immune cells in pan-cancer and lung cancer clinical samples, we identified a critical role for *RRM2* in LUAD. *RRM2* is a rate-limiting enzyme which is involved in DNA synthesis and repair. It also plays a vital role in many critical cellular processes, such as cell proliferation, invasiveness, migration, angiogenesis, and aging (56). In breast cancer, *RRM2* overexpression in cancer cells promotes the formation and invasion of 3D colonies (57). In liver cancer (58), *RRM2* can inhibit hypertrophy by stimulating GSS to synthesize GSH. In LUAD, *RRM2* has been determined to have an independent prognostic significance. *RRM2* expression levels have significant correlations with B cells, CD4+ T cells, and neutrophil infiltration (59). We also determined that *RRM2* was highly related to the CDK family of proteins. As Cyclin-dependent kinases 4 and 6 (*CDK4/6)* are important regulators of cell cycle and inhibit the proliferation of regulatory T cells (60). Therefore, *RRM2* could also be involved in cell cycle regulation. Our findings further confirmed the relationship of *RRM2* with immunity and metabolism in LUAD. Moreover, our study provided a base and theoretical support for exploring the immunotherapy of LUAD.

## Conclusions

In this study, we first identified the vital role of immune-related metabolic genes in lung adenocarcinoma’s immune and clinicopathological aspects. We clustered three subtypes of LUAD based on immune-related metabolic genes and these subtypes exhibited different survival and immune status. We identified nine genes i.e., *TK1、TCN1、CAV1、ACMSD、HS3ST2、HS3ST5、AMN、ADRA2C*, and *ACOXL* that were used to construct a prediction model. Finally, *RRM2* was determined as a promising metabolism checkpoint for LUAD and explored its close relationship with the CDK family of proteins. Our results are therefore, helpful for the study of immunotherapy and immune-related metabolic genes in LUAD.

## Data Availability Statement

The datasets presented in this study can be found in online repositories. The names of the repository/repositories and accession number(s) can be found in the article/**Supplementary Material**. All analyses were performed using R version 3.6.3.

## Ethics Statement

The studies involving human participants were reviewed and approved by the First Affiliated Hospital of Zhengzhou University approved the study (Ethics number: 2020-KS-HNSR188). The patients/participants provided their written informed consent to participate in this study.

## Author Contributions

FL: Conceptualization of the study. CH and LQ: Analyzed the data. PL: Drafted the manuscript. JS: Conducted the experiments and revised manuscript. GZ: Guided on the quality of the research. All authors read and approved the submission of the final manuscript.

## Conflict of Interest

The authors declare that the research was conducted in the absence of any commercial or financial relationships that could be construed as a potential conflict of interest.

## Publisher’s Note

All claims expressed in this article are solely those of the authors and do not necessarily represent those of their affiliated organizations, or those of the publisher, the editors and the reviewers. Any product that may be evaluated in this article, or claim that may be made by its manufacturer, is not guaranteed or endorsed by the publisher.

## Abbreviations

TCGA, The Cancer Genome Atlas; KEGG, Kyoto Encyclopedia of Genes and Genomes; GO, Gene Ontology; DEGs, differentially expressed genes; LUAD, Lung adenocarcinoma; ssGSEA, single sample gene set enrichment analysis; TME, tumor microenvironment; PPI, protein-protein interaction.
