# Supplementary material for: Comprehensive Analysis of Immune-Related Metabolic Genes in Lung Adenocarcinoma
Source: Front Endocrinol (Lausanne). 2022 Jul 8;13:894754. doi: 10.3389/fendo.2022.894754 (PMC9309246; doi:10.3389/fendo.2022.894754)
Supplement: Supplementary file 1 [file Table_1.docx]

**Supplementary table 1. Primers used in RT-PCR analysis.**

| Name | Sequence (5′-3′) |
| --- | --- |
| RRM2-F | CACGGAGCCGAAAACTAAAGC |
| RRM2-R | TCTGCCTTCTTATACATCTGCCA |
| HMMR-F | AGAACCAACTCAAGCAACAGG |
| HMMR-R | AGGAGACGCCACTTGTTAATTTC |
| PFKP-F | GACCTTCGTTCTGGAGGTGAT |
| PFKP-R | CACGGTTCTCCGAGAGTTTG |
| TCN1-F | CCCCTAGTGGGGCTCTTACT |
| TCN1-R | CAGAGGTTTTAGGCGGATGTAG |
| TK1-F | GCCAAAGACACTCGCTACAG |
| TK1-R | CCCCTCGTCGATGCCTATG |
| CDK2-F | GTACCTCCCCTGGATGAAGAT |
| CDK2-R | CGAAATCCGCTTGTTAGGGTC |
| CDK4-F | TCAGCACAGTTCGTGAGGTG |
| CDK4-R | GTCCATCAGCCGGACAACAT |
| CDK6-F | TCTTCATTCACACCGAGTAGTGC |
| CDK6-R | TGAGGTTAGAGCCATCTGGAAA |
| CDK8-F | AGCCAGTTCAGTTACCTCGG |
| CDK8-R | CTGTGCAACACCCAGTTAGCA |
| GAPDH-F | GGAGCCAAAAGGGTCATCACTC |
| GAPDH-R | GAGGGGCCATCCACAGTCTTCT |
