# Supplementary material for: Comprehensive Analysis of Immune-Related Metabolic Genes in Lung Adenocarcinoma
Source: Front Endocrinol (Lausanne). 2022 Jul 8;13:894754. doi: 10.3389/fendo.2022.894754 (PMC9309246; doi:10.3389/fendo.2022.894754)
Supplement: Supplementary file 2 [file DataSheet_1.docx]

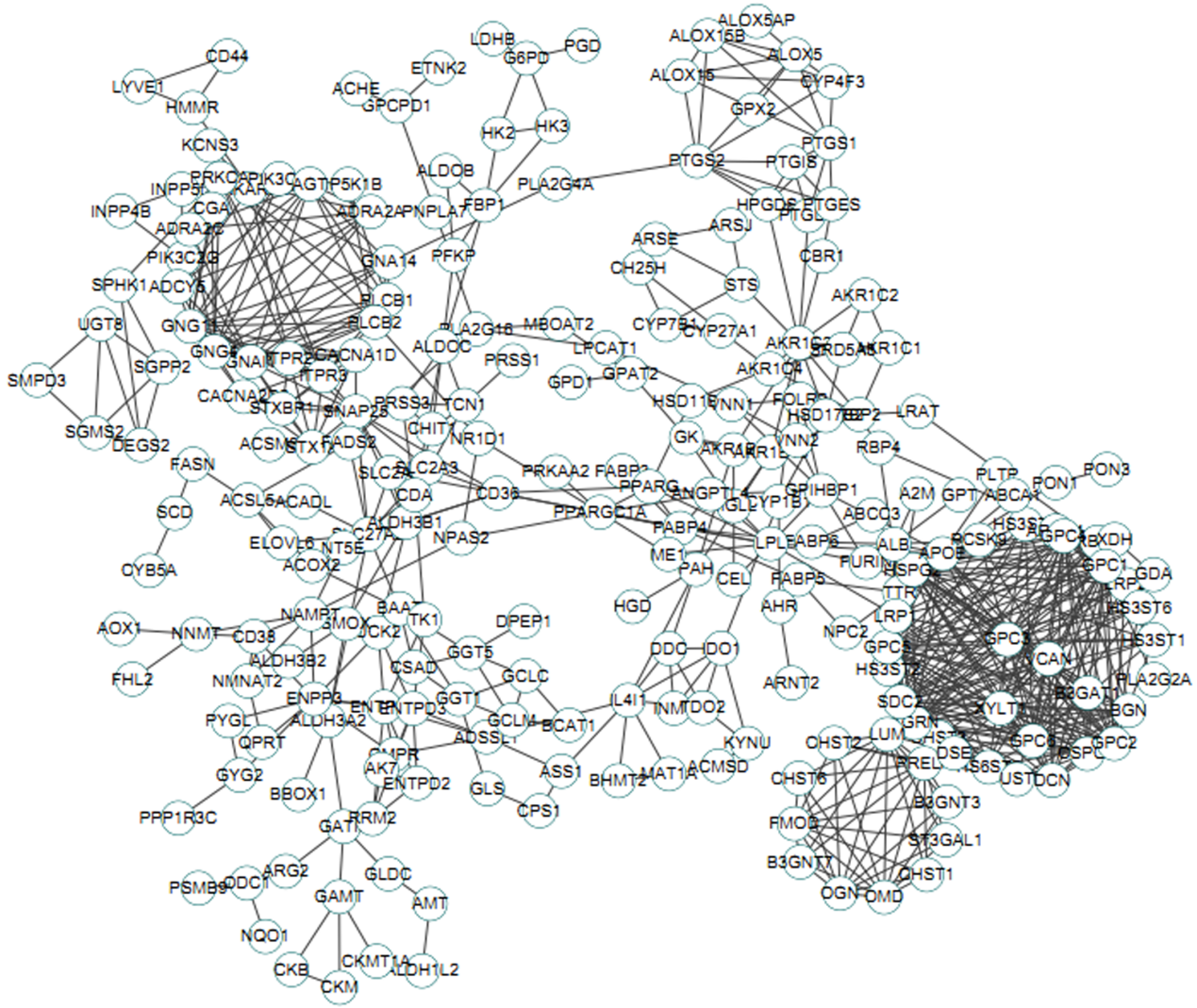


**Supplementary Figure 1: PPI network.** Interaction network constructed with the nodes was shown. Interaction confidence value >0.9.

**
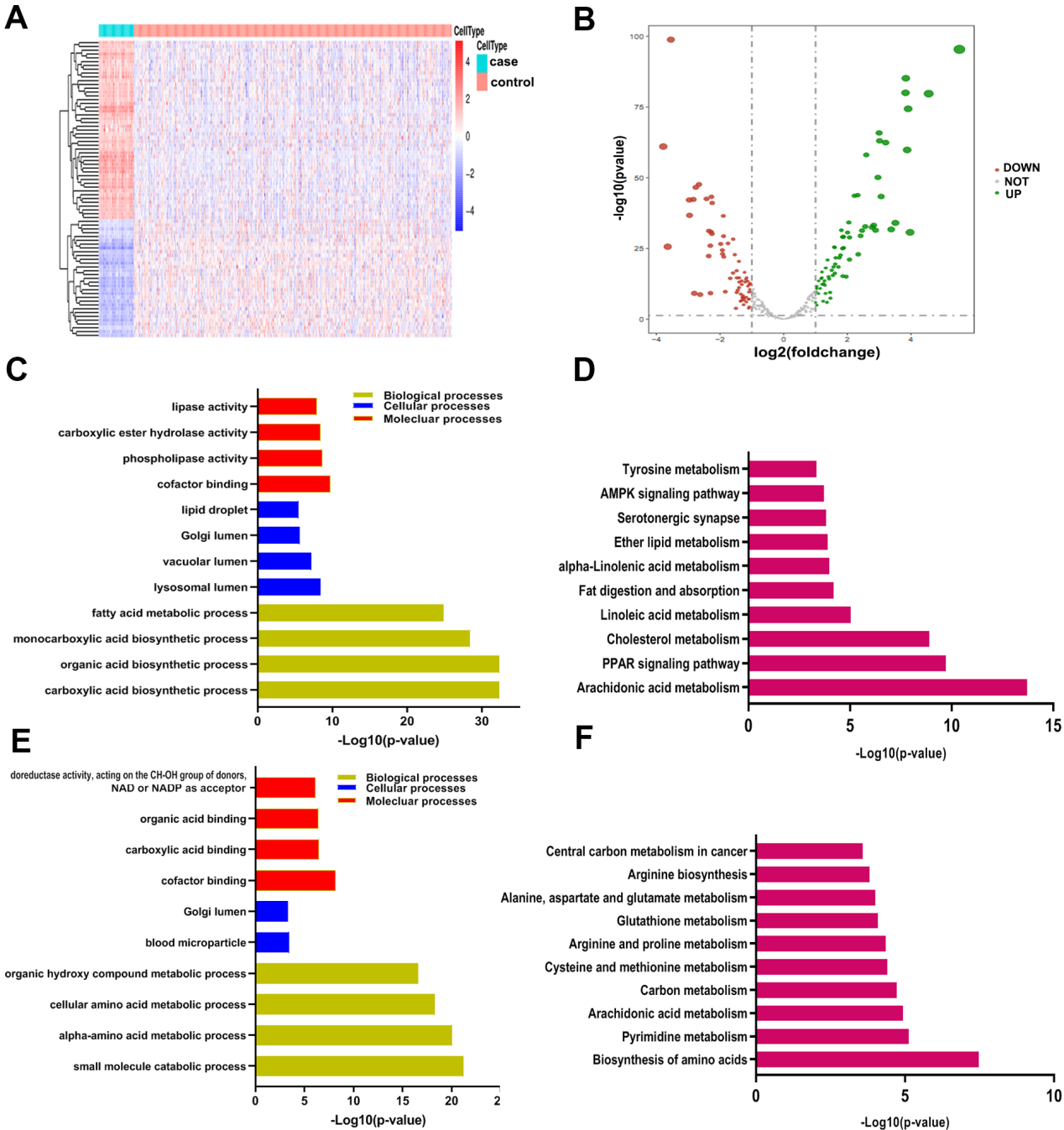
**

**Supplementary Figure 2: GO and KEGG of DEGs in lung cancer and adjacent tissues.** (A) Heat map of the 141 DEGs with |log2fold change | > 1, P <0 .05 were shown. Red color indicated higher expression; blue color indicated lower expression; (B) Volcano plot of 141 DEGs was shown. Red color indicated upregulation with |log2fold change| > 1, P <0 .05; blue color indicated downregulation with |log2fold change| > 1, P <0 .05; black color indicated unchanged genes, (C-F) GO and KEGG enrichment analysis on 141 differential genes. BP: biological process; CC: cellular component; MF: molecular function; KEGG: Kyoto Encyclopedia of Genes and Genomes.


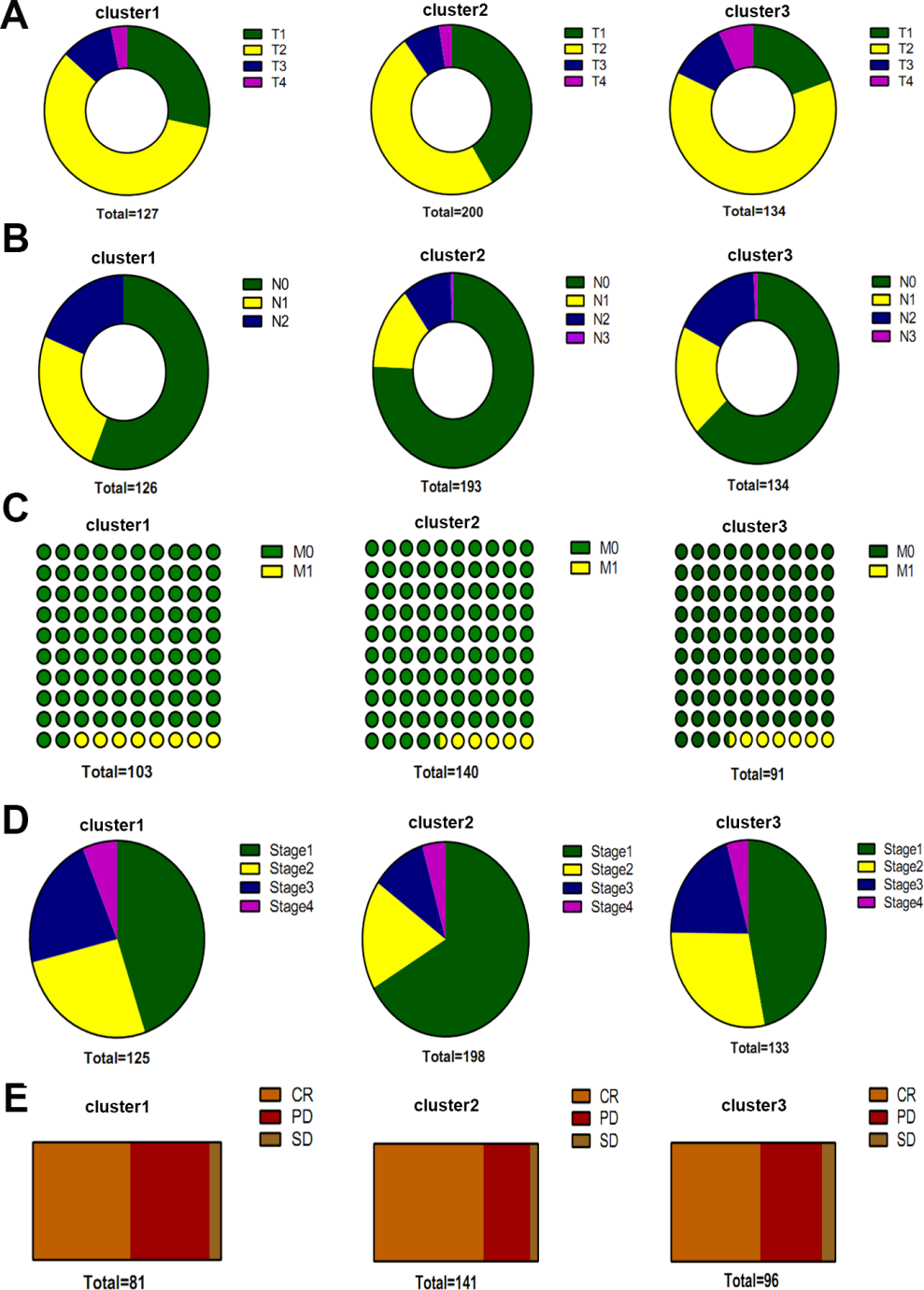


**Supplementary Figure 3: Clinicopathological characteristics in different clusters.** Different clinicopathological parameters among different clusters were shown; (A-I) TNM classification; (J-L) tumor stage, and (M-O) drug response characteristics were shown.


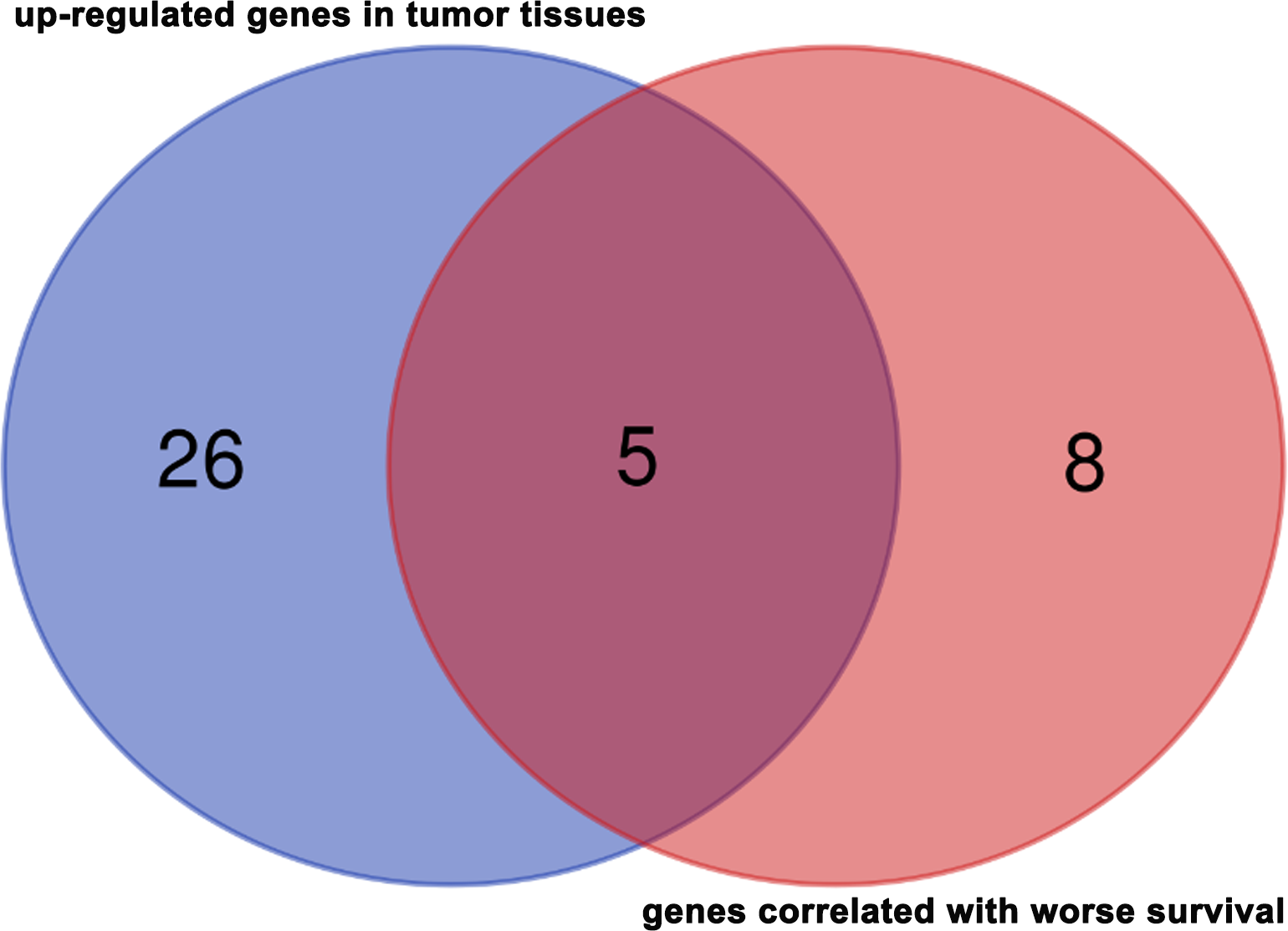


**Supplementary Figure 4: Venn diagram of genetic intersection.**


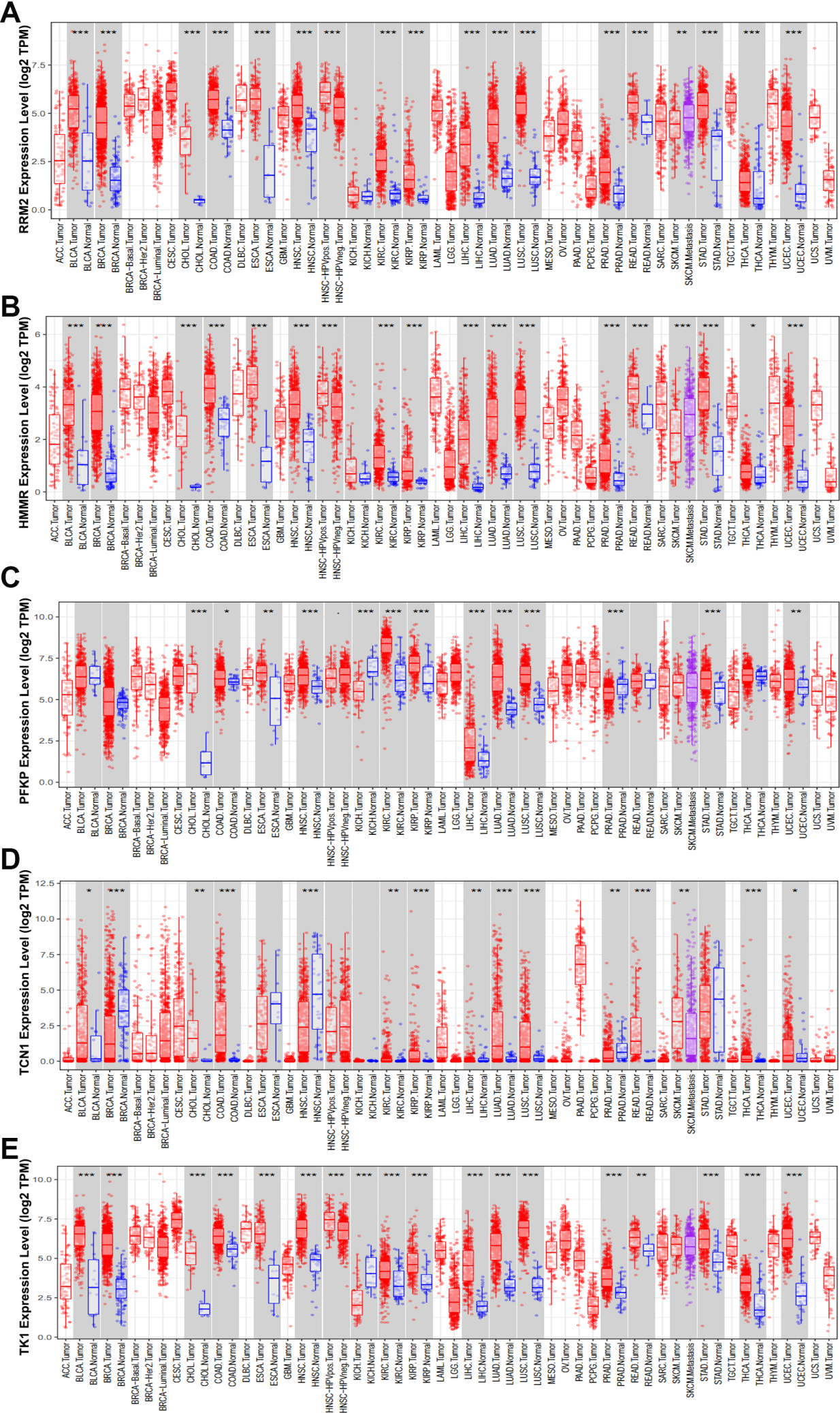


**Supplementary Figure 5: differential expression of gene map in Pan-cancer.** (A-E) Expression levels of five hub genes were compared (*RRM2, HMMR, PFKP, TCN1* and *TK1*) between tumors and normal tissues.


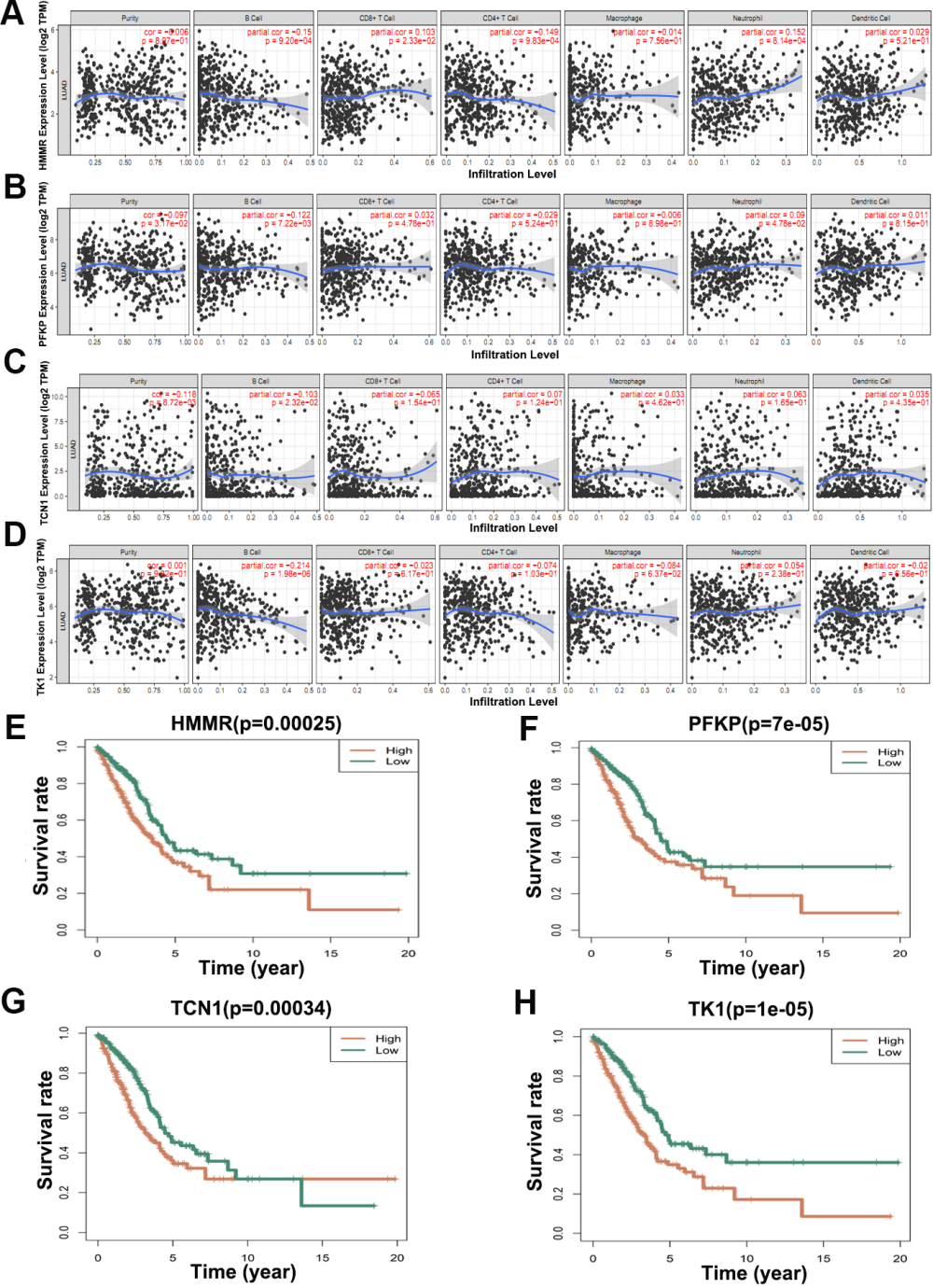


**Supplementary Figure 6: Correlation and survival maps of genes and immune cells.** (A-D) Correlation map of *HMMR, PFKP, TCN1* and *TK1* with seven kinds of immune cells was shown; (E-H) The survival time of *HMMR, PFKP, TCN1* and *TK1* expressions was shown.


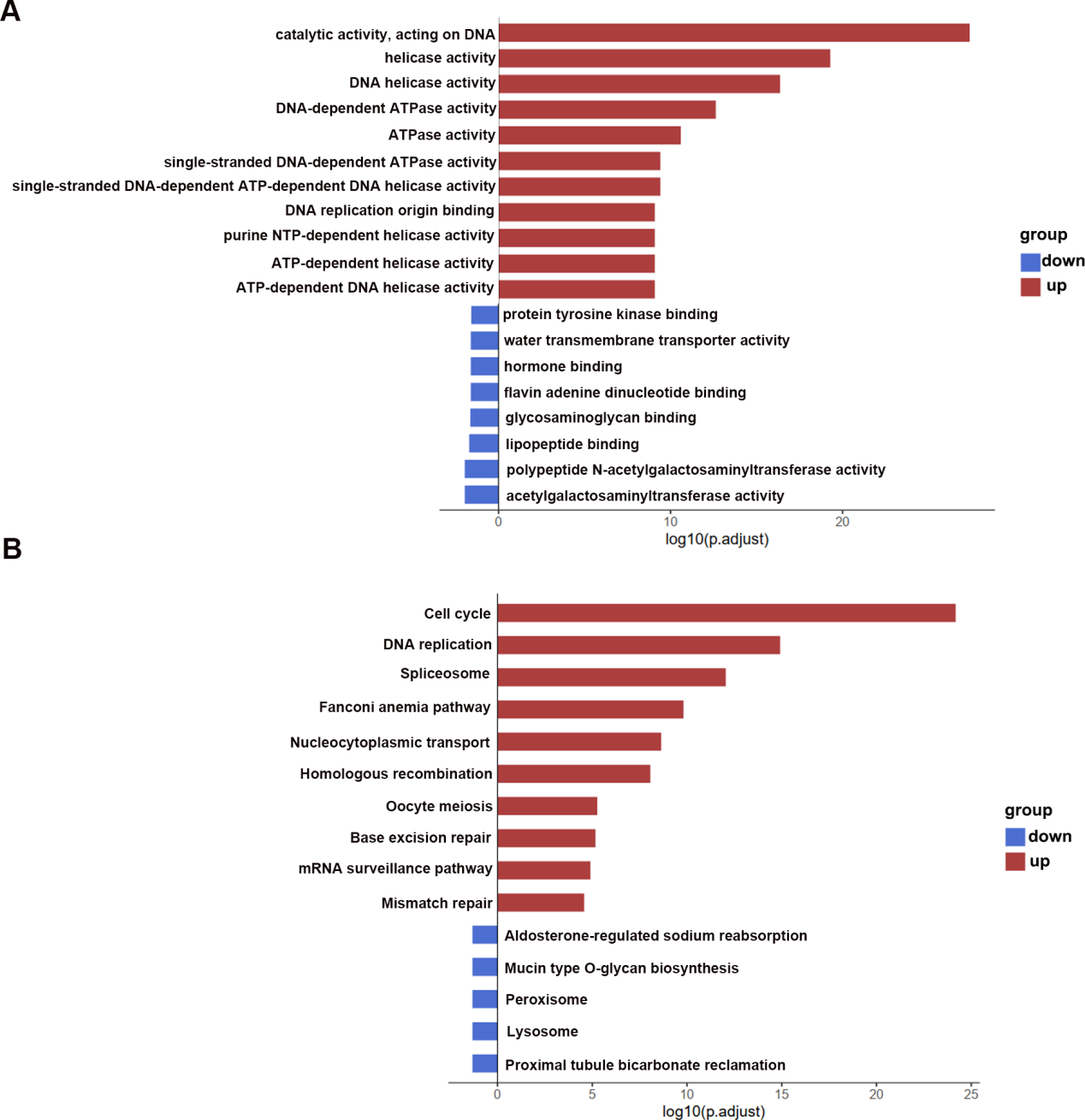


**Supplementary Figure 7:** GO and KEGG analysis of the genes that were correlated with *RRM2*.
